# Age related changes in T cell mediated immune response and effector memory to Respiratory Syncytial Virus (RSV) in healthy subjects

**DOI:** 10.1186/1742-4933-7-14

**Published:** 2010-10-20

**Authors:** Maria Grazia Cusi, Barbara Martorelli, Giuseppa Di Genova, Chiara Terrosi, Giuseppe Campoccia, Pierpaolo Correale

**Affiliations:** 1 Department of Molecular Biology, Microbiology Section, University School of Medicine, V.le Bracci, 1, 53100 Siena, Italy; 2 Blood Bank, Policlinico "S. Maria delle Scotte", V.le Bracci, 1, 53100 Siena, Italy; 3 Department of Pharmacology "Giorgio Segre" Institute, Siena University School of Medicine, V.le Bracci, 1, 53100 Siena, Italy

## Abstract

Respiratory syncytial virus (RSV) is the major pathogen causing respiratory disease in young infants and it is an important cause of serious illness in the elderly since the infection provides limited immune protection against reinfection. In order to explain this phenomenon, we investigated whether healthy adults of different age (20-40; 41-60 and > 60 years), have differences in central and effector memory, RSV-specific CD8+ T cell memory immune response and regulatory T cell expression status. In the peripheral blood of these donors, we were unable to detect any age related difference in term of central (CD45RA^-^CCR7^+^) and effector (CD45RA^-^CCR7^-^) memory T cell frequency. On the contrary, we found a significant increase in immunosuppressive regulatory (CD4^+^25^+^FoxP3^+^) T cells (T_reg_) in the elderly. An immunocytofluorimetric RSV pentamer analysis performed on these donors' peripheral blood mononuclear cells (PBMCs), *in vitro *sensitized against RSV antigen, revealed a marked decline in long-lasting RSV specific CD8+ memory T cell precursors expressing interleukin 7 receptor α (IL-7Rα), in the elderly. This effect was paralleled by a progressive switch from a Th1 (IFN-γ and TNF-α) to a Th2 (IL-10) functional phenotype. On the contrary, an increase in T_reg _was observed with aging. The finding of T_reg _over-expression status, a prominent Th2 response and an inefficient RSV-specific effector memory CD8+ T cell expansion in older donors could explain the poor protection against RSV reinfection and the increased risk to develop an RSV-related severe illness in this population. Our finding also lays the basis for new therapeutic perspectives that could limit or prevent severe RSV infection in elderly.

## Introduction

Respiratory Syncytial Virus (RSV) is a single-stranded negative sense RNA virus that causes severe lower respiratory tract infection in infants and young children worldwide [1,2]. Although reinfections occur frequently during life and everyone encounters RSV from an early age on, many immunocompromised individuals and elderly people develop severe RSV complications [3-6]. RSV specific CD8 T cell responses are involved in the clearance of the virus and recovery from infection [7-9], therefore, an efficient CTL response appears to be necessary to prevent and/or control the reinfection in humans [10-12]. Nevertheless, primary RSV infection does not confer a prolonged and efficient immune-protection and for this reason reinfections are commonly recorded [13]. The biological reasons that sustain this poor protection and the high susceptibility to develop severe RSV infection in the elderly are not completely known. However, the results of preclinical studies in mouse models suggest that RSV infection may generate a direct inhibition of T cell activation and suppression of the effector activity in the antigen specific T cells [8,11-15]. RSV infection, in particular, seems to be able to disregulate RSV-Ag specific CTL expression and to suppress memory development selectively in the respiratory tract. A rapid loss of RSV specific memory CD8+ T cells in the lungs after infection has been noted in mice [16,17]. Much less information is available on RSV interaction with immune system in humans, in fact very few studies have provided significant insight into the development of the specific immunological memory against this virus [10,18]. We believe that it is important to understand the mechanisms underlying the long maintenance of RSV specific memory cells in humans, because these could explain the basis of the higher susceptibility to reinfection in the elderly.

We therefore, designed our study based on the knowledge that viral infections generally produce a long-lasting immunity, whose entity is critically related to an efficient expansion of antigen specific effector memory CD8^+ ^T cell precursors not counter-balanced by an exaggerate immune-suppressive regulatory T cell (T_reg_) response [19,20]. It has been recently shown that in the memory phase specific CD8+ T cells express interleukin-7 receptor α (IL-7Rα+) and show enhanced proliferation in response to homeostatic signals, indicating that these cells develop into long-lasting memory cells which would make IL-7Rα a useful marker for cells destined to become memory cells [21-24]. Moreover, age associated changes occurring in T cell subsets and functions in humans could play an important role in the immune response against some viruses, such as RSV, for the elderly. To this aim, we analysed the expression of circulating RSV-specific CD8^+ ^T cell subsets and immune-suppressive regulatory T cells in the peripheral blood of healthy volunteers belonging to three different age groups. The first one ranging from 20 to 40 years (group 1); the second one, from 40 to 60 years (group 2) and the third from people older than 60 years (group 3). In this study, we also evaluated the frequency of different RSV specific CD8^+ ^T lymphocyte subsets expressing central memory (T_CM_), (CD45RA^-^CCR7^+^), effector memory (T_EM_) (CD45RA^-^CCR7^-^) and effector memory RA45^+ ^(T_EMRA_) (CD45RA^+^CCR7^-^) phenotype [25,26] in donors' peripheral blood lymphocytes (PBLs) before and after *in vitro *sensitization to RSV. In particular, we carried out a pentamer assay directed to an immune-dominant RSV peptide epitope with class I HLA specific binding motifs [27], on PBMCs unstimulated, or *in vitro *stimulated with an RSV peptide antigen, in order to mimic the natural occurrence of reinfection *in vivo*.

## Materials and methods

### Study population

Forty-five healthy participants aged 20-65 years, with no history of pulmonary diseases and normal lung function, were enrolled among healthy blood donors. They were divided in three groups: the first included people aged 20-40 years (n = 13, median age, 37 years), the second, people aged 41-60 years (n = 18, median age, 51 years) and the third people aged > 60 years (n = 14, median age, 63 years). All subjects were HLA-A(*)02.01^+ ^positive by standard serological and molecular techniques performed by the hematology laboratory ("Santa Maria alle Scotte" Hospital, Siena, Italy) and were seropositive to RSV.

All the participants provided informed consent and the study was approved by the Medical Ethics Committee of the Academic School of Medicine of the University of Siena.

### Analysis of T cell populations by multiparameter flow cytometry after *in vitro *Ag stimulation

Peripheral blood mononuclear cells (PBMCs) were isolated from heparinized blood of healthy HLA-A(*)02.01 volunteers using a lymphocyte separation medium gradient (Pharmacia Biotech, Uppsala, Sweden), as previously described [28].

PBMCs were cultured in 6-well plates (2 × 10^6 ^cells/ml) in 3 ml of RPMI medium (InVitrogen) supplemented with 10% human serum type AB (Cambrex Bio-Science, Walkersville Inc., MD, USA) in the presence of 30 μg/ml of the N-RSV1 (KMLKEMGEV aa.137) peptide (GeneScript Corporation, New Jersey, USA) with high HLA-A(*)02.01 binding motifs [27], for 5 days. Cells were then cultured for two days in the presence of IL-2 (10 IU/ml), thereafter they were stimulated with peptide for further 24 h. As negative control, cells were stimulated with an unspecific HLA-A(*)02.01 peptide, PTR-4 (TSTTSLELD) [29], derived from a protein produced by prostate carcinoma.

Cells were then incubated with antibodies against PE conjugated anti-human CD8 (STEMCELL Technologies Köln, Germany), FITC conjugated anti-human CD45RA (eBioscience San Diego, CA, USA), PE conjugated anti-human CCR7 (BD Biosciences Pharmigen, San Diego, CA, USA), FITC conjugated anti-human CD127 (IL-7Rα) (eBioscience San Diego, CA, USA), FITC conjugated anti-human Bcl-2 oncoprotein (Dako cytomation, Glostrup, Denmark), cocktail of FITC conjugated anti-human CD4 & PE conjugated anti-human CD25 (eBioscience San Diego, CA, USA), APC conjugated anti-human FoxP3 (eBioscience San Diego, CA, USA) for flow-cytometric analysis, using a FACSCalibur flow cytometer and CellQuest Pro Software (BD Biosciences). For Tregs analysis, cells were stained for CD4, CD25 expression and permeabilized before staining with anti- FoxP3 (eBioscience, clone PCH101). The percentages of FoxP3^+ ^CD4^+ ^and CD25^high ^CD4^+ ^cells were in gated CD3^+ ^T cells.

### RSV specific antibody detection

The RSV specific antibody titre was evaluated in the serum samples of the subjects enrolled in this study. Sera were analysed by indirect immunofluorescence assay and neutralization assay. Briefly, HEp-2 cells (ATCC - CCL-23) were infected with hRSV (type A, Long strain) at a moi of 1. Three days post-infection, cells were fixed with cold acetone/methanol (1:1) for 10 minutes. Serum samples were diluted from 1:10 to 1:160 in PBS and the slides were incubated in a humidified chamber at 37°C for 1 hour. After washing with phosphate-buffer, the slides were incubated with a fluorescein-conjugated goat anti-human immunoglobulin G diluted in PBS (diluted 1:32) (Sigma, Milan, Italy). A commercial positive serum (Virostat, Portland, ME, USA) was used as control for the assay. The slides were examined under a UV microscope at magnification of × 400. The same sera were also analysed for the presence of RSV specific neutralizing antibodies. The assay was performed as previously described [30]. Neutralizing antibodies were expressed as the reciprocal of the last dilution exhibiting 50% of residual infectivity on Hep-2 cells infected by RSV.

### Determination of antigen-specific CD8+ T cell frequency by pentamer

The cells analysed for pentamer, CD45, CCR7, and CD127 markers were previously purified on column (Miltenyi Biotec Inc. Bergish Gladbach, Germany) for isolation of CD8+ T lymphocytes from PBMCs (2×10^6^). CD8+ T cells were incubated with R-PE-conjugated HLA-A*0201 pentamer loaded with the N-RSV1 (ProImmune, Oxford, UK) to mark RSV specific cells, and CD127-fluorescein isothiocyanate antibody (eBioscience San Diego, CA, USA), according to the manufacturer's instructions. For negative control, PBMCs isolated from a non-matching HLA were used. Fifty thousand events/sample were analysed by flow cytometry.

### Cytokine assay

About 100 μl of 2 × 10^6 ^unfractionated PBMCs per ml in a complete RPMI 1640 plus 10% FCS were cultured with 30 μg/ml of RSV peptide or phytohemagglutinin (PHA) (Sigma, Milan, Italy) (5 μg/ml) in a 96 well flat-bottomed plate. Control wells received cell suspension only. After 48 h in culture, cell-free supernatants were harvested and stored at -80°C until use. IL-10, IFN-γ, and TNF-α were tested according to the manufacturer' instructions by using the specific commercially available assays (IFN-γ, Southernbiotech, Birmingham, USA, and TNFα, SABiosciences Corporation Frederick, MD USA). Samples were diluted 1:1 with PBS, 1% BSA and 0.05% Tween 20 and incubated with mAb-coated capture for 2 h at room temperature. After washing, the plates were incubated with labeled anti-human cytokine antibody for 2 h. The concentration of cytokines in samples was determined according to the standard curve. IFN and TNF-α produced by CD8+ T cells were also tested by intracellular cytokine staining for flow cytometry analysis in unstimulated and stimulated lymphocytes (as previously described), after 15 h treatment with brefeldin A (10 μg/ml final) (Sigma, Milan. Italy).

### Statistical analysis

The between-mean differences were statistically analyzed using Stat View statistical software (Abacus Concepts, Berkeley, CA). Comparisons within three unpaired groups were made using the Kruskal-Wallis test, and a two-tailed Mann-Whitney test was used when comparing only two unpaired groups. A *P*-value of < 0.05 was considered statistically significant.

## Results

### Determination of the frequency of naïve, central memory (CM) and effector memory (EM) CD8^+ ^T cells

Based on the differential expression of CD45RA and CCR7, we evaluated the frequency of T naïve (CD45RA^+ ^CCR7^+^), T_CM _(CD45RA^-^CCR7^+^), T_EM _(CD45RA^- ^CCR7^-^) and T_EMRA _(CD45RA^+^CCR7^-^) [25,31,32] on purified CD8^+ ^T cells isolated from the PBMCs of healthy subjects belonging to three restricted age cohorts [group 1, 20-40 years; group 2, 40-60 years; and group 3, > 60 years]. Our analysis revealed a similar frequency of these T cell subsets with no intergroup differences either when PBMCs were analysed at the baseline and when they were *in vitro *stimulated with RSV antigen (Table 1).

**Table 1:** CD8^+^Naïve, T_CM_, and T_EM _cells frequency for gated lymphocytes before and after *in vitro *stimulation with N-RSV1 peptide (30 μg/ml) in healthy blood donors within the three groups of age

Subjects	Naïve Baseline	Naïve Ag stimulated	T_CM_ Baseline	T_CM _Ag stimulated	T _EM_ Baseline	T _EM _Ag stimulated	T _EMRA_ Baseline	T _EMRA _Ag stimulated
20-40 y	40 ± 15	35 ± 11	3.5 ± 2	2.7 ± 1.2	20.4 ± 8	28 ± 7	32.7 ± 9	35.6 ± 8
41-60 y	38.7 ± 15	40.5 ± 24	3 ± 1.4	5 ± 4	21 ± 10	26 ± 17	37.8 ± 10	28.7 ± 14
> 60 y	37.7 ± 13	37 ± 10.5	4.2 ± 1.9	5 ± 3.4	19.6 ± 10	25 ± 12	33.7 ± 10	32.1 ± 12

Values are given as percentage frequency ± SD

### Determination of RSV peptide specific CD8^+^CD127^- ^and CD8^+^CD127^+ ^(memory) T cell response

**Figure 1:**
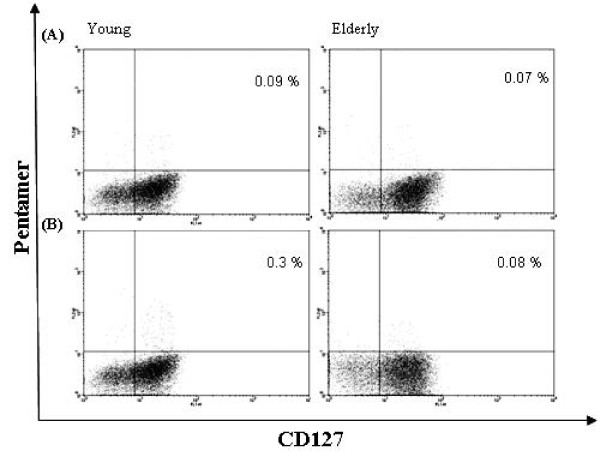
**Detection of RSV^+^pentamer^+^CD127^+^CD8^+ ^T cells in peripheral blood**. Prototypes of dot plots for young (20 years) or old (> 60 years) HLA-A2.1 positive participants, at baseline (A) and after stimulation with N-RSV1 (B). Shown is a dot plot of isolated CD8^+ ^T cells fluorescence for RSV1- pentamer (R-PE) and anti-CD127 (FITC) gated on forward-scatter and side-scatter parameters. The frequency of double positive cells is expressed as percentage of total CD8^+^T cells in the quadrant.

The results of recent studies indicate that the expression of different homing receptors may be correctly used to recognise different T_CM _T_EM _and T_EMRA _subsets [23,24,31-35]. In this context, antigen specific CD8^+ ^T cells which express interleukin-7 receptor α (CD127^+^) show enhanced proliferation in response to homeostatic signals and most likely develop into long-lasting memory cells. IL-7 stimulation is crucial for memory T cell survival and proliferation, and a high IL-7Rα/CD127 expression warrants these cells with the ability to persist for long period in the absence of antigen stimulation [21,36]. CD127 may therefore, be considered as a useful marker to identify effector lymphocytes precursors destined to differentiate into memory cells [22]. In our study we have evaluated the expression of CD127 in RSV-peptide specific CD8^+ ^T cells identified by pentamer binding in healthy subjects. These cells were examined before and after *in vitro *sensitization with RSV peptide (N-RSV1). The fraction of pentamer^+ ^cells gated on total CD8^+ ^cells, was used to measure the frequency of RSV-specific memory T cells. We recorded no differences among the different subjects when the frequency of RSV specific/CD8^+^CD127^- ^ranging from 0.04% to 1.2% was examined at baseline (*P *> 0.05). No difference was similarly observed among the age groups when the PBMCs were *in vitro *stimulated with RSV peptide (*P *> 0.05) (Table 2). We also analysed the frequency of RSV specific/CD8^+^CD127^+ ^of the same samples and, again, no age related difference was observed at baseline (*P *> 0.05) in all the three groups (Table 2). On the contrary, when the analysis was performed on the same PBMCs *in vitro *stimulated with RSV peptide, we observed significant age related changes in the expression of CD127^+ ^immune-effectors. In fact, we recorded a significant decline of CD8^+^CD127^+ ^T cells in the stimulated PBMCs of the oldest group (group 3) (Figure 1B), which was not observed in the PBMCs of the other two youngest groups (*P *= 0.04) (Table 2) indicating that PBMCs of the oldest subjects did not respond to the *in vitro *antigen stimulation with RSV peptide. This behaviour was not observed in the PBMCs obtained from people of groups 1 and 2 which, after the *in vitro *Ag stimulation, significantly increased the frequency of CD8^+^/pentamer^+^/CD127^+ ^T cells (*P *= 0.004 and 0.003 respectively), indicating that there was an effective expansion in antigen specific T cells with long lasting memory (Figure 1B).

**Table 2:** CD8^+ ^Pentamer^+ ^CD127^+ ^T cells and CD8^+ ^Pentamer^+ ^CD127^- ^T cell frequency of gated lymphocytes before and after *in vitro *stimulation with N-RSV1 peptide (30 μg/ml) in healthy blood donors within the three groups of age.

Subjects	CD8^+ ^Pentamer^+ ^CD127^+^ Baseline	CD8^+ ^Pentamer^+ ^CD127^+^ Ag Stimulated	CD8^+ ^Pentamer^+ ^CD127^-^ Baseline	CD8^+ ^Pentamer^+ ^CD127^-^ Ag Stimulated
20-40 y	0.07 ± 0.03	0.24 ± 0.1	0.033 ± 0.02	0.04 ± 0.02
41-60 y	0.09 ± 0.05	0.19 ± 0.1	0.04 ± 0.02	0.07 ± 0.03
> 60 y	0.08 ± 0.03	0.07 ± 0.04	0.02 ± 0.02	0.028 ± 0.01

(50.000 events/samples were analysed)

Values are given as percentage frequency ± SD

#### Bcl-2 expression

IL7-R signalling may promote lymphocyte survival necessary for long living memory T cells, protecting these cells from multiple pro-apoptotic stimula and inhibitory feedback [22,37]. IL-7 binding to CD127 leads, in particular, to an increased expression of anti-apoptotic molecules, such as Bcl-2 [37]. In order to understand whether RSV-Ag stimulation could promote apoptotic deletion of RSV specific memory T cells in the elderly, we evaluated Bcl-2 expression in the PBMCs of the three groups of subjects after antigen stimulation *in vitro*. Our results failed to show significant differences in IL-7 induced Bcl-2 expression among the different groups, although, Ag stimulation of the PBMCs of the oldest donors showed a trend to a reduced expression of this molecule (from 0.11% ± 0.03 to 0.08% ± 0.02), which did not achieve statistical significance (*P *> 0.05) in comparison with those from the youngest individuals (data not shown).

### Analysis of immune-suppressive regulatory T cells

An immune-suppressive regulatory T cells feed-back response is frequently associated with infections and chronic inflammation with the specific task of controlling the entity of immune-response thus avoiding autoimmunity and dangerous overreactions [38-41]. We therefore, analysed the amounts of T_reg_s expressing a CD4^+^CD25^High+^FoxP3^+ ^immunophenotype in the PBMCs derived from subjects of the three groups of age. T_reg _expression was in particular, evaluated at baseline and after *in vitro *RSV Ag stimulation. The ratio Treg-cells/CD4^+ ^lymphocytes at baseline, increased in the oldest group in comparison with the other two groups (P = 0.001) (Table 3) while these differences were lost upon antigen stimulation. In fact, T_reg_s' frequency upon Ag stimulation resulted similar in all the samples, without any significant difference with the corresponding values obtained at baseline.

**Table 3:** T_reg _cell percentage ± SD on total CD4^+ ^cells before and after *in vitro *stimulation with N-RSV1 peptide (30 μg/ml) in healthy blood donors within the three groups of age

Subjects	T_regs_ Baseline	T_regs_ Ag Stimulated
20-40 y	1.9 ± 0.6	2.6 ± 0.7
41-60 y	1.8 ± 0.4	1.7 ± 0.3
> 60 y	3.2 ± 1	2.3 ± 0.7

### Th1 and Th2 functional phenotype

In order to investigate whether there was a different functional response to RSV in lymphocytes of the different age related subjects, we investigated the production of cytokines correlated with a Th1 or Th2 cell phenotype in response to RSV peptide (NRSV-1) *in vitro *stimulation. To this aim, PBMCs were incubated with the RSV peptide for 48 hours, before the supernatant were analysed for the expression of IFN-γ, TNF-α (indicative of a Th1 response) and IL-10 (indicative of a Th2 response). In these experiments fresh medium was used as a negative control, while PHA was used as a positive control for *in vitro *stimulation. In our experiments, we observed a lower Th1 response to RSV-1 in PBMCs in the elder donors (group 3). Lower levels of IFN-γ (47.3 ± 4.2 pg/ml) were produced by lymphocytes of the oldest subjects (> 65 years) after *in vitro *stimulation with NRSV-1 peptide in comparison with the other adults (*P *= 0.001) (Table 4). TNF-α, which is considered an important factor associated with pro-inflammatory and Th1 responses, clearly showed a trend to decrease with the age, reaching very low values at the baseline of the oldest group (*P *= 0.009) (Table 4). When TNF-α production was evaluated after the *in vitro *stimulation with RSV peptide, we did not find any change in the supernatant of PBMCs of the oldest people (group 3) (*P *= 0.2), while we observed a significant decrease of it in the supernatant of the stimulated PBMCs of the first and second group of age (*P *= 0.026 and 0.004, respectively) (Table 4). Similar results were obtained analysing intracellular IFN-γ and TNF-α produced by CD8+ lumphocytes. We also evaluated the production of IL-10, a cytokine representing a Th2 functional phenotype, in the PBMCs of these donors. We recorded an age related progressive increase in the level of this cytokine which was not modified by antigen stimulation and was maximal in older patients (Table 4). Intracellular expression of IL-10 did not provide suitable results.

**Table 4:** Cytokine expression after *in vitro *stimulation with N-RSV1 peptide (30 μg/ml) of PBMCs of healthy blood donors within the three groups of age

Subjects	IFN-γ	IL-10	TNF-α
**Ag**	**- +**	**- +**	**- +**

1° group (20-40 years)	57.4 ± 5.8 68.9 ± 3.4	44.7 ± 9.6 29.1 ± 3.1	895.1 ± 2.3 114.1 ± 4.5
2° group (41-60 years)	58.5 ± 4.6 72.4 ± 2.7	64.7 ± 3.6 133.9 ± 8.2	646.5 ± 32.6 49.1 ± 3.8
3° group (> 60 years)	45.3 ± 4.8 47.3 ± 4.2	252.2 ± 12.6 267.8 ± 23	123 ± 8.7 36 ± 5.2

Values are given in pg/ml ± RSV

### Humoral response to RSV

This study analysed the baseline humoral response to RSV in the subjects of the three groups of age. It was shown that the neutralizing antibody level was quite low or absent in elderly subjects in spite of the presence of specific antibodies revealed by the immunofluorecence test. The mean antibody titre appeared higher in the elderly, indicating that some subjects had probably been reinfected by RSV. However, these individuals appeared not to be protected by specific neutralizing antibodies, since these were asbsent in their serum samples (Table 5). Consequently, it seems very important to test the neutralizing antibody titres in patients for evaluating their potential protective level against RSV.

**Table 5:** RSV specific antibody response tested by neutralization and immunofluorescence assays

Groups of age	GMT ± SD by NT	GMT ± SD by IFA
20-40 years	22	52
40-60 years	20	34
> 60 years	2,5	80

GMT: geometric mean titre

## Discussion

RSV infection causes severe bronchiolitis in children and severe pulmonary disease in elderly individuals [1-5]. In this study we tried to understand the immunological mechanisms which could make elderly more susceptible to RSV reinfection and at risk of serious respiratory disease. Although after primary infection children develop a CTL response that clears the virus and should protect against reinfection for many years, it is still not clear why this virus is associated with a high reinfection rate and high morbidity in the frail elderly or in people with cardiopulmonary disease [3-6].

The progressive age related decline in immune memory has been proposed as a fundamental mechanism for this phenomenon. It is essential that the RSV specific memory T cells persist after primary infection to keep enduring immunological protection. The mechanisms behind the development of long-lasting memory cells are incompletely understood but, recently, it was shown that IL-7Rα^+ ^effector cells are able to survive antigen deprivation and develop into long living memory CD8^+ ^T cells [22,36]. To this aim, we characterized the CD8^+ ^CD127^+ ^long-lasting memory cells specific to RSV by pentamer in healthy subjects of three different age groups. This analysis was performed by evaluating the immunological parameters in human PBMCs at baseline and after *in vitro *stimulation with a specific RSV peptide to partially mimic the natural events occurring after *in vivo *reinfection.

However, it is worthy to note that no cytokines were added to the stimulated lymphocytes in order not to interfere with the microenvironment and cell proliferation. Therefore, although environment factors such as IL-7 or IL-15 may be fundamental to expand memory cells [37], these were not considered in this study to rule out possible artefacts and to exclude the risk of using the same amounts of cytokines for all the subjects without considering the variability due to age.

In the present study, we analysed the different CD8^+ ^memory cell populations and focused on the long-lasting memory subset in the presence or absence of RSV antigen. For PBMCs stimulation, we used an RSV peptide derived from the nucleocapsid antigen, able to stimulate human HLA-A(*)02.01^+ ^peripheral blood mononuclear cells, as previously shown [27]. It is worthy to note, however, that a number of virus specific T cells may be missed because they do not recognize the epitopes contained in the pentamer. On the basis of classical characterization of T_CM, _T_EM, _and T_EMRA _[25], we did not find differences among the three age groups, nor were differences based on age found for specific CD8^+^/CD127^- ^T cells labelled with RSV pentamer. On the contrary, a clear decline of RSV specific CD8^+^/CD127^+ ^cells was evident after antigen stimulation in the oldest group, indicating that this specific memory population tends to diminish with age and reaches very low levels when the subject is newly exposed to the virus. Moreover, a proliferative induction of this population is triggered in PBMCs of the young after Ag stimulation, while no induction is evident in the oldest group. This data indicates that RSV specific memory cells are able to re-expand following the antigenic boosting, but this capability decreases in size with increasing age. This was probably due to a down-regulation of T cell receptor (TCR) after some reinfections or because IL-7 level, which is fundamental for rejuvenating T cell immunity and improving the survival of memory T cells, declines with age [42]. Moreover, Bcl-2, which is an anti-apoptotic molecule that is critically involved in the IL-7 mediated cell survival, appears to be less expressed, in the elderly, in RSV specific CD8^+^CD127^+ ^memory cells which then differentiate in effector cells, indicating that a Bcl-2 down-regulation occurs with the age. Although other studies have shown that influenza virus and RSV specific cells retained a high IL-7Rα expression in the lung [43], hypothesizing that these cells were activated by their cognate antigen and might rely on IL-7, this data does not consider the age of the patients and does not explain whether these specific cells are recruited in the lung from the circulation. Therefore, trying to understand this evident decrease of RSV specific memory CD8^+ ^T cells in the elderly, we analysed the frequency of CD4^+^CD25^+^FoxP3^+ ^T cells and defined natural regulatory (T_reg_) cells which have a negative regulatory effect on immune responses [38,39,44,45]. CD8^+ ^T-cell effector functions and proliferation are often dampened by negative regulation by T_reg _cells which play a role in regulation of adaptive immune response to several viruses [46]. In this study, the increase of T_reg _cells in the elderly, matched with a parallel increase of IL-10, that is an important suppressive cytokine, produced by a large number of immune cells in addition to the antigen-driven IL-10-producing regulatory and the naturally occurring suppressor CD4^+ ^T cells [41]. In fact, the amount of IL-10 expressed by PBMCs without Ag stimulation, in people > 60 years, was very high (252,2 ± 12,6 pg/ml) in comparison with the level of the youngest subjects (Table 4). The inhibitory effect of T_reg_s on Th1 response [47] was also demonstrated by a diminished IFN-γ production in older subjects in comparison with the younger and by a shift from Th1 cytokines (including IFN-γ and TNF-α) to Th2 cytokines (including IL-10) with aging. In this study, we observed that the Th1 response was also characterized by a marked decrease of the proinflammatory cytokine, TNF-α, in the elderly, and that this tended to further diminish after Ag stimulation. Consequently, it is likely that CTL response and the effector activity mediated by CD8^+ ^T cells are no more efficient to combat the virus. Although recent studies have shown evidence that RSV immune response is mainly compartmentalized in the lung [18], the data presented here confirm a decrease of specific CD8^+ ^memory cells with the age in the peripheral blood of healthy subjects. This could be due, in particular, to the high frequency of T_reg_s in old people that could partly suppress the specific immune response and to a weakness of the Th1 response, determining a greater frequency of RSV reinfection during the elderly. Moreover, since infection can and does occur in the presence of circulating antibodies, a balanced immune response including RSV specific neutralising antibodies would be necessary for maintaining protective immunity in the host. These results show, in addition, that there is a decline of RSV specific protective humoral response in elderly which, as in this case, could be partly due to the increase of T_reg_s. In fact, these cells are able to suppress the proliferation not only of CD8^+ ^but also CD4^+ ^lymphocytes [41], that interacting with B cells specific for peptide antigens can generate a T cell dependent antibody response. In this context, we believe that the antigen hierarchy may be completely subverted with a potential deletion of viral epitopes specific for immune effector precursors which are critical for an efficacious protection against viral reinfection. In fact, in our study, we have observed a higher amount of circulating anti-RSV antibodies with lower protecting activity in elderly persons. Early studies on influenza virus suggested that hypothetical suppressogenic epitopes can be responsible for induction of antigen-specific T "suppressor " cells [48]. Why these regulatory cells exert their action on specific cells is still unclear and needs further study. In light of these results, manipulation of T_reg_s may be critical to enhance immune responses in the aged and new strategies aimed at limiting T_reg_s would be useful for prevention of severe RSV infection in frail, susceptible people.

## Competing interests

The authors declare that they have no competing interests.

## Authors' contributions

MGC conceived and designed the experiments. GDG and CT performed the experiments. MGC and PC analyzed the data. GC contributed materials and participated in the study coordination. MGC wrote the paper. All authors read and approved the final manuscript.
